# Tributyrin (CoreBiome^®^) enhances butyrate levels and modulates the gut microbiota, barrier function, and immune response *in vitro*

**DOI:** 10.3389/fnut.2025.1712993

**Published:** 2025-11-24

**Authors:** Cindy Duysburgh, Lynn Verstrepen, Lieven Van Meulebroek, Massimo Marzorati

**Affiliations:** 1 ProDigest, Zwijnaarde, Belgium; 2 CMET, University of Ghent, Ghent, Belgium

**Keywords:** butyrate, gut barrier integrity, immunomodulation, gut microbiome, postbiotic, SHIME, tributyrin

## Abstract

**Background/objectives:**

Oral butyrate is unstable during upper gastrointestinal tract (GIT) transit and very little reaches the colon. Tributyrin, a butyrate precursor, resists gastric acid and is converted to butyrate by pancreatic lipases. This study aimed to quantify tributyrin stability during upper GIT passage and to uncover the effects of tributyrin supplementation on the human gut microbiome and cellular responses.

**Methods:**

*In vitro* upper GIT simulations were used to evaluate the stability of a capsule and softgel formulation of tributyrin (CoreBiome^®^). The effects of tributyrin supplementation on the human gut microbiome and cellular responses were evaluated using the Simulator of the Human Intestinal Microbial Environment (SHIME^®^) model and Caco-2/THP1 co-cultures.

**Results:**

The upper GIT simulations showed that 40.9 and 48.7% of the tributyrin dose administered via the capsule or softgel, respectively, was hydrolyzed to butyrate in the small intestine; 59.1 and 51.3% remained stable and was available to enter the colon. Using the SHIME^®^ model, it was shown that 3 weeks of daily tributyrin supplementation increased butyrate levels and enhanced the abundance of several bacterial species, including *Bifidobacterium* spp. and *Akkermansia mucinophila*. Metabolic impacts on the gut microbiome were also observed. Assessment of cellular responses revealed that tributyrin fermentation had a protective effect on the intestinal barrier and exerted immunomodulatory properties.

**Conclusion:**

Enhanced butyrate concentrations and beneficial impacts on the gut microbial community composition were observed in an *in vitro* simulation of the human intestinal environment, suggesting that tributyrin could be considered as a solid alternative to butyrate supplementation.

## 1 Introduction

Postbiotics have been defined by the International Scientific Association of Probiotics and Prebiotics as a “preparation of inanimate microorganisms and / or their components that confers a health benefit to the host” (1). Postbiotics have shown benefits similar to those of probiotics, but in the absence of live microorganisms. Some characteristics ascribed to postbiotics include protective effects against pathogens, strengthening the intestinal barrier, and immunomodulation (2).

Butyrate is an important postbiotic metabolite produced by the bacteria in the colon. It is a major source of energy for colonocytes (3, 4) and is associated with a healthy intestinal barrier as well as immunomodulatory and anti-inflammatory effects. It supports a healthy gut microbiome (5), making it important for human health. Considering its many beneficial effects, butyrate supplementation has been evaluated in clinical studies in patients with a variety of conditions, including inflammatory bowel disease, pediatric obesity, lead-induced neuroinflammation, cancer, and chronic obstructive pulmonary disease (6–12). When supplemented orally, the majority of butyrate is absorbed in the small intestine, and relatively little butyrate reaches the colon (5, 13, 14). Tributyrin is a butyrate precursor that resists gastric acids to allow more butyrate to reach the colon. Once in the intestine, pancreatic lipases break the bond between the three molecules of butyrate and one molecule of glycerol, allowing a more effective delivery of butyrate within the gastrointestinal environment (15, 16).

Studies evaluating tributyrin as a feed supplement have found positive impacts on the intestinal health of multiple species, including cows, chickens, and pigs (17–21). A study with obese mice who were fed a high fat diet demonstrated that tributyrin supplementation resulted in less body weight gain, improved insulin responsiveness and glucose metabolism, and reduced adipose tissue inflammation compared with mice who received a placebo (22). Tributyrin supplementation in antibiotic-treated mice provided protection against antibiotic-induced reductions in short-chain fatty acid (SCFA) levels and resulted in reduced intestinal tissue expression of inflammatory mediators and increased expression of tight junction proteins compared with mice who received a placebo, suggesting that tributyrin has a protective effect on the intestinal barrier (23). Tributyrin supplementation has also been reported to increase non-rapid-eye movement sleep in mice (24). A pilot study in healthy humans reported that oral supplementation with a tributyrin complex for 21 days resulted in reduced levels of high-sensitivity C-reactive protein, suggesting a possible anti-inflammatory effect (25). Considering the evidence of beneficial effects following tributyrate supplementation along with the improved stability in the digestive tract compared to butyrate, tributyrin may be a more effective alternative to butyrate supplementation (26–28).

*In vitro* models that accurately simulate the physiological conditions of the human gastrointestinal tract (GIT) allow researchers to collect samples from different regions without the need for invasive procedures. This facilitates more detailed and mechanistic studies of the GIT and the effect of test products on different aspects of gastrointestinal function. Such detailed studies would not be possible in humans due to the need for invasive sample collection procedures. While animal studies may act as a surrogate, they are imperfect models in that the colonic background and physiology of the GIT vary considerably between humans and non-human animals. Studies using *in vitro* models of the human GIT can be considered complementary to clinical trials.

During the current study, we assessed the stability of two formulations of tributyrin, a capsule formulation and a softgel, during passage through the upper GIT *in vitro*. Furthermore, we evaluated the effect of tributyrin supplementation on the composition and metabolic activity of the gut microbiome and cellular responses using the validated Simulator of the Human Intestinal Microbial Ecosystem (SHIME^®^) model combined with *in vitro* co-culture cell assays.

## 2 Materials and methods

### 2.1 Study design

In this study, several *in vitro* experiments were conducted to assess different outcomes related to tributryin. First, *in vitro* upper GIT simulations using the SHIME^®^ model were employed to evaluate the release of tributyrin and the conversion to butyrate. The model evaluated its degradation products during passage through the human upper GIT under fasted conditions. Second, the effects of tributyrin supplementation on the community composition and activity of the gut microbiota from three healthy human donors were assessed using the Triple-L-SHIME^®^ model. Finally, the effects of tributyrin fermentation on intestinal barrier integrity and immune markers were assessed using an *in vitro* Caco-2/THP1-Blue™ co-culture model.

### 2.2 Fecal samples

Fecal samples were collected from three healthy adult donors (donor A: M, 31y; donor B: M, 26y; donor C: M, 33y). The donors had no history of chronic disease, had not taken any antibiotics in the 4 months prior to sample donation, and had a western diet pattern. In an anaerobic environment, phosphate buffered saline was added to the freshly acquired samples which were homogenized to create a slurry. The samples were briefly centrifuged to remove large particles and an equal volume of optimized in-house cryoprotectant [modified from Hoefman et al. (29)] was added. Finally, the samples were flash frozen and placed at −80 °C prior to the experiment, the samples were defrosted and added to the reactors. The collection and use of the fecal samples was performed in accordance with the protocol approved by the Ethics Committee of the University Hospital Ghent (reference number ONZ-2022-0267; approved on 29 July 2022).

### 2.3 Tributyrin test products

Two formulations of tributyrin (CoreBiome®) were evaluated in the upper GIT simulation. The first was a capsule formulation containing tributyrin powder, resulting in a total concentration of 300 mg tributyrin per capsule. The second was a softgel formulation containing liquid tributyrin, resulting in a total concentration of 450 mg tributyrin per softgel. For the Triple-L-SHIME^®^ study, pure liquid tributyrin was dosed directly into the proximal colon in the doses shown to reach the colon, circumventing the need for tributyrin degradation during the small intestinal transit. The tributyrin capsules, softgels, and pure liquid were provided by Compound Solutions Inc. (Carlsbad, CA, USA).

### 2.4 *In vitro* simulations

#### 2.4.1 Upper GIT simulation

The upper GIT simulation comprised gastric and small intestinal incubation in a fasted state. It was based on the consensus protocol developed within COST Action InfoGest (30) with modifications that included the use of a dynamic pH profile that more closely mimics *in vivo* conditions. The gastric and small intestinal incubations were conducted subsequently in the same reactor. A capsule sinker was used to insert the capsules/softgels into the reactor (2 capsules per reactor [total of 600 mg tributyrin powder] or 1 softgel per reactor [450 mg liquid tributyrin]) at the start of the gastric incubation. The samples were then incubated for 45 min (37 °C with stirring) in nutrient-depleted gastric fluid (3 g/L mucin, 7 mM KCl, 50 mM NaCl, 0.17 mM phosphatidylcholine, 4,000 U/mL pepsin [Chem Lab, Zedelgem, Belgium]) at pH 2.0. To transition to the small intestinal phase, the reactor pH was increased from 2.0 to 5.5 over 5 min using 0.1 M sodium bicarbonate. During the 3 h small intestinal incubation, the pH was further increased to 7.0 (gradual increase from 5.5 to 6.5 during the first hour, gradual increase to 7.0 during the second hour, and remained constant at 7.0 during the final hour). At the same time, 3.33 mM bovine bile extract, 15.4 TAME U/mL trypsin (Carl Roth, Karlsruhe, Germany), and 3.8 BTEE U/mL chymotrypsin (Carl Roth) were added. Samples were collected at 0, 15, 30, and 45 min during the gastric phase and at 30, 60, 90, 120, 150, and 180 min during the small intestinal phase, then immediately frozen for further analysis. All experiments were performed in biological triplicate.

#### 2.4.2 Triple-L-SHIME^®^

A Triple-L-SHIME^®^ setup was used for this study (Supplementary Figure S1). This setup is based on the original SHIME^®^ model described by Molly et al. (31) with some modifications to allow for the simulation of three investigational arms in parallel. In this case, the effects of test product supplementation on the fecal microbiota collected from three healthy donors were assessed. To allow for the additional test conditions, the setup included two colon regions, the proximal colon and the distal colon, as compared to the three regions in the standard SHIME^®^ setup. The proximal colon reactors had a pH of 5.7–5.9, a retention time of 20 h, and a volume of 500 mL and the distal colon reactors had a pH of 6.6–6.9, a retention time of 32 h, and a volume of 800 mL. Throughout the experiment, the reactors were fed three times daily with standard L-SHIME^®^ nutritional medium (15.6 g/L PDNM001B, ProDigest, Belgium). Following inoculation of the reactors with the fecal samples, the microbial community was allowed to differentiate and adjust to the conditions of the local environment for 2 weeks (stabilization period), thereby reaching a stable microbial community (quality criterium: >80%, with 96.3% stability reached in the current study). During the control period (6 consecutive weekdays, spread over 2 weeks) samples from the reactors were analyzed to determine the baseline microbial community composition and activity in the different colonic regions. The control period was followed by a three-week treatment period where pure liquid tributyrin was added directly into the proximal colon at a dose representative of the *in vivo* target dose of 300 mg/day. The actual dosing was based on the finding from the upper GIT simulation that 59.1% of the administered tributyrin reached the colon (i.e., 59.1% of the target dose was supplied).

#### 2.4.3 Caco-2/THP1-blue™ co-culture model

Co-culture experiments were performed using Caco-2 (HTB-37; American Type Culture Collection) and phorbol-12-myristate-13-acetate differentiated THP1-Blue™ cells (InvivoGen; San Diego, CA, USA) as previously described (32, 33). Briefly, sterile filtered (0.22 μM) colonic suspensions collected during the control and treatment week 3 (TR3) periods were added to the co-cultures and incubated for 24 h (37 °C, 5% CO_2_, humidified atmosphere). The basolateral medium was then discarded, and the cells were stimulated with 500 ng/mL ultrapure lipopolysaccharide (LPS; *Escherichia coli* K12, InvivoGen) for 6 h (37 °C, 5% CO_2_, humidified atmosphere). Samples from the colonic incubations were used in the cell co-culture assay as technical triplicates.

### 2.5 Study readouts

#### 2.5.1 Tributyrin stability

Tributyrin stability was assessed using the samples collected during the upper GIT simulation. Levels of tributyrin and butyrate were assessed via liquid–liquid extraction using acetonitrile as solvent (1:1) followed by capillary gas chromatography coupled with a flame ionization detector according to the methods of De Boever et al. (34). The limit of quantification (LOQ) for tributyrin and butyrate was equal to 0.1 mM and 0.25 mM, respectively. Values below the LOQ were replaced by zero.

#### 2.5.2 Microbial activity

Samples for assessment of microbial activity were collected three times per week from the control period onwards from both colonic regions. Levels of SCFAs (acetate, propionate, butyrate) and branched-chain fatty acids (BCFA) were assessed via liquid–liquid extraction followed by capillary gas chromatography coupled with a flame ionization detector according to the methods of De Boever et al. (34). Lactate was quantified using the Enzytec™ kit (R-Biopharm, Darmstadt, Germany), according to manufacturer’s instructions. An AQ300 Discrete Analyzer (SEAL Analytical, WI, USA) was employed to assess ammonium levels using the indophenol blue method (35). Samples from the control and TR3 periods were compared for both the proximal and distal colon.

#### 2.5.3 Untargeted metabolic fingerprinting

Metabolomics of the gut ecosystem is concerned with the comprehensive analysis of metabolites, providing a direct functional read-out of the physiological status of the intestinal microbiome. Untargeted metabolic fingerprinting is thereby used as in first-line segregation of samples based on distinctive metabolic fingerprints, by detecting *m/z* features and their relative abundances across samples. Samples for untargeted metabolic fingerprinting were collected once per week from the control period onwards from both colonic regions. Untargeted metabolic fingerprints were obtained for each sample using the Laser-Assisted Rapid Evaporative Ionization Mass Spectrometry (LA-REIMS) platform (36, 37). Briefly, samples were thawed (4 °C) then vortexed for 1 min (400 rpm, 20 °C) and 200 μL was transferred into wells of a 96-well plate. The LA-REIMS platform comprised a MID infrared laser system (OpoletteTM HE2940, OPOTEK, LLC, Carlsbad, CA, USA) and a Xevo™ G2-XS Quadrupole Time-of-Flight mass spectrometer (Waters Corporation, Wilmslow, UK) operated in negative ionization mode with an *m/z*-scan range of 50–1,200 Da applied.

#### 2.5.4 Metagenomic analysis

Samples for metagenomic sequencing were collected three times per week during the control period and TR3 from both colonic regions. DNA was extracted using the CTAB DNA extraction method (38). Kneaddata v0.10.0 was employed for quality filtering, trimming, and host decontamination (human genome [hg37] of raw reads with the params:-SLIDINGWINDOW:5:22 MINLEN:100 AVGQUAL:22) to determine the taxonomic classification of shallow shotgun metagenomic samples. The obtained quality filtered reads were then analyzed with Kraken2 v2.1.3 (confidence threshold, 0.1) and Bracken v2.9 (read threshold, 50) using the Genome Taxonomy Database (R214 along with Refseq genomes from fungi + protozoan + virus) containing ± 100,000 species for taxonomic classification. Flow cytometry set to a high flow rate was used to quantify the total number of bacterial cells in each sample (BD Accuri C6 Plus Flow Cytometer; BD Biosciences, Franklin Lakes, NJ, USA). The SYTO channel was set to a threshold level of 700 to separate bacterial cells from signal noise and medium debris. Populations were determined by setting appropriate parent and daughter gates. This allowed for the conversion of the metagenomics data from relative abundances to absolute abundances by multiplying relative abundances in a sample with the total cell count.

#### 2.5.5 Intestinal permeability and cytokine responses

Samples for assessment of effects on intestinal permeability and cytokine responses were collected once at the end of the control and treatment period from both colonic regions. To assess permeability in the Caco-2/THP1-Blue™ co-cultures, the transepithelial electrical resistance (TEER) of the Caco-2 cells was measured at baseline (i.e., empty insert) and after 24 h incubation with supernatants collected from the colonic reactors. Basolateral supernatants collected following the 6 h LPS stimulation were used to assess cytokine levels (interleukin [IL]-6, IL-1β, IL-10, and tumor necrosis factor [TNF]-α) which were quantified using a Luminex® multiplex (ThermoFisher Scientific, Waltham, MA, USA) according to manufacturer’s instructions.

### 2.6 Statistical analysis

Levels of released tributyrin and butyrate in the stomach and small intestinal simulations were compared using a two-tailed homoscedastic Student’s t-test. Levels of SCFAs, lactate, BCFA, and ammonium in colonic supernatants from the Triple-L-SHIME^®^ experiments were compared using two-tailed homoscedastic Student’s t-tests for the individual donors, and using paired Student’s t-tests across donors. TEER values and cytokine levels obtained in the Caco-2/THP1 experiment were compared using two-way analysis of variance (ANOVA) with Sidak’s multiple comparisons tests in GraphPad Prism version 9.4.1 (GraphPad Software, San Diego, CA, USA). A *p*-value of <0.05 was considered statistically significant.

For analysis of the metabolic fingerprinting (LA-REIMS), data obtained from the Triple-L-SHIME^®^ experiment were normalized and subjected to multivariate statistical analysis using SIMCA 17 (Sartorius, Germany). During pre-processing, data were log-transformed to induce normal distributions and unit variance scaling (1/standard deviation) to standardize the range of signal intensities. The natural patterning of samples and identification of potential outliers (based on the Hoteling’s T^2^ criterion) was accomplished using Unsupervised Principal Component Analysis (PCA-X). Orthogonal Partial Least Squares Discriminant Analysis (OPLS-DA) was used to differentiate samples by experimental conditions in a supervised fashion. The validity of the OPLS-DA models was verified by the quality parameter Q^2^(Y) (≥0.5), cross-validated ANOVA (*p*-value < 0.05), and permutation testing (*n* = 100) (39).

Metagenomics data obtained from the Triple-L-SHIME^®^ experiment were used for the following analyses. Beta-diversity was analyzed using Discriminant Analysis of Principal Components (DAPC) with *a priori* defined groups (40). TreeclimbR analysis (41) were used to identify the taxa most likely to explain the differences between the control and treatment period. Bacterial enrichments were considered statistically significant if they had a –log(*p*-value) > 1.3. Volcano plots were used to visualize the statistical significance versus magnitude of change for each taxon. This effectively classifies taxa into four different categories based on abundance in compared conditions (i.e., control versus treatment): (1) not significant and not biologically relevant (−2 < log_2_ fold change [FC] < +2, and –log_10_[*p*-value] < 1.3), (2) biologically relevant, but not statistically significant (log_2_FC < −2 or log_2_FC > +2, and –log_10_[*p*-value] < 1.3), (3) statistically relevant, but not biologically relevant (−2 < log_2_FC < +2, and –log_10_[*p*-value] > 1.3), and (4) biologically and statistically significant (log_2_FC < −2 or log_2_FC > +2, and –log_10_[*p*-value] > 1.3). TreeclimbR analysis was run using treeclimbR v0.1.5 and edgeR v3.42.421. Benjamini-Hochberg multiple testing correction was used, and the alpha-level was set at 0.05.

## 3 Results

### 3.1 Release and stability of tributyrin in the upper GIT

**Figure 1:**
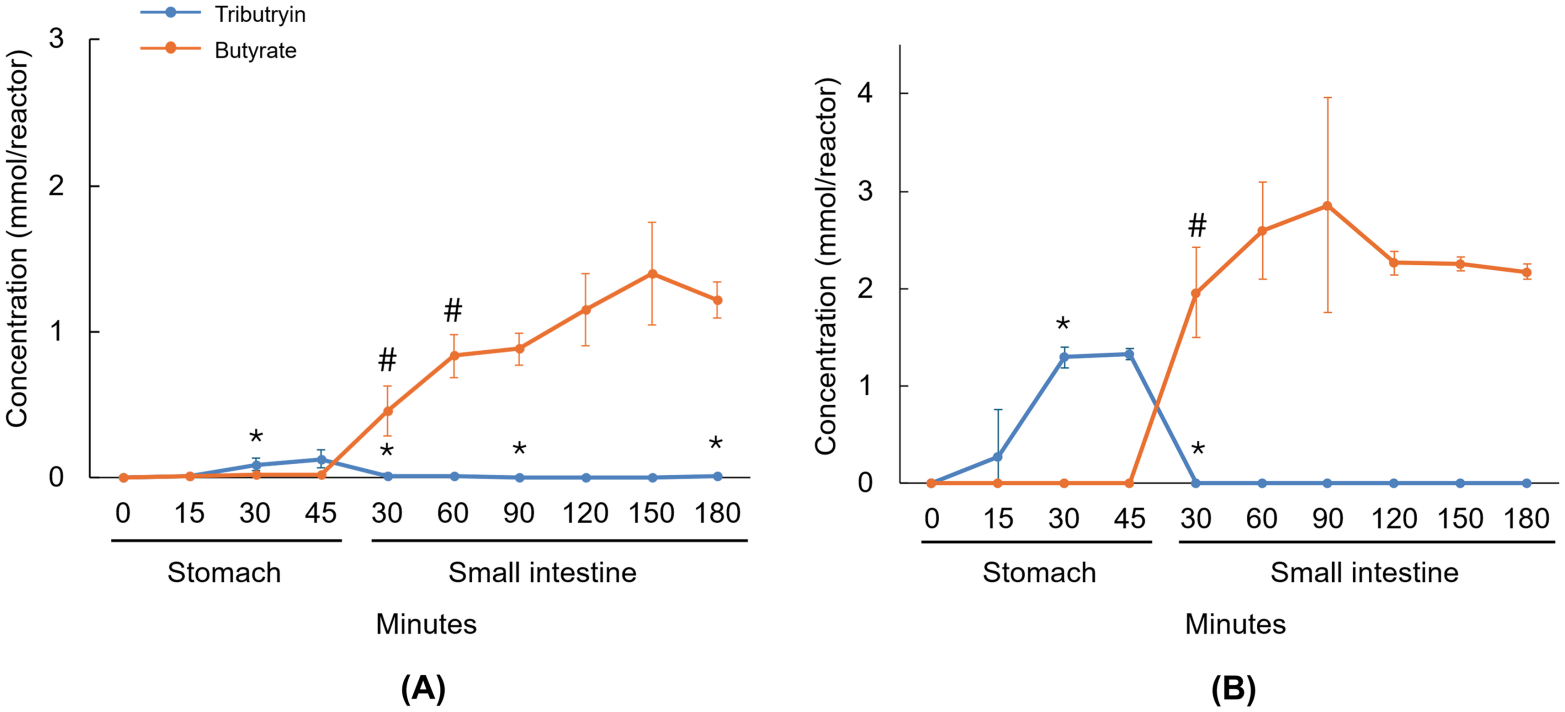
Line graphs visualizing the average concentration of tributyrin and butyrate during passage through the upper GIT under fasted conditions for the capsule **(A)** and softgel **(B)** formulations of tributyrin. Administered concentrations of tributyrin were 300 mg and 450 mg for the capsule and softgel formulation, respectively. Data are plotted as mean ± standard deviation (*n* = 3). *p < 0.05 versus the preceding timepoint for tributyrin measurements. #*p* < 0.05 versus the preceding timepoint for butyrate measurements. *p*-values were determined using a two-tailed homoscedastic Student’s t-test. GIT, gastrointestinal tract; SI, small intestine; ST, stomach.

The levels of tributyrin and its degradation product, butyrate, during the stomach and small intestinal incubations are shown in Figure 1. During gastric transit, full dissolution of the capsule formulation was not obtained yet, though an initial release of tributyrin was observed during stomach incubation (reaching significance after 30 min compared to the previous timepoint), which decreased to very low levels at the start of the small intestinal incubation (Figure 1A). Butyrate levels increased at the start of the small intestinal incubation (reaching significance at 30 min and 60 min compared to previous timepoints), indicating that tributyrin was hydrolyzed to butyrate in the small intestine. With the softgel formulation, full dissolution was again not observed during gastric transit, though tributyrin levels significantly increased 30 min after initiating the stomach incubation and remained high at 45 min, decreasing to low levels after the small intestinal incubation was initiated (showing a significant reduction after 30 min compared to the previous timepoint) (Figure 1B). Evidence of tributyrate degradation (i.e., increased butyrate levels) was observed at the start of the small intestinal incubation (reaching significance after 30 min compared to the previous timepoint). At the end of the small intestinal incubation, 1.22 (±0.13) and 2.17 (±0.08) mmol butyrate was present with the capsule and softgel formulations, respectively, demonstrating that 40.9% (±4.2%) and 48.7% (±1.9%) of the initial tributyrin dose of each of the test products was hydrolyzed to butyrate in the small intestine and that 59.1% (±4.2%) and 51.3% (±1.9%) of the administered tributyrin remained stable and was available to enter the colon.

### 3.2 Microbial metabolic activity

**Figure 2:**
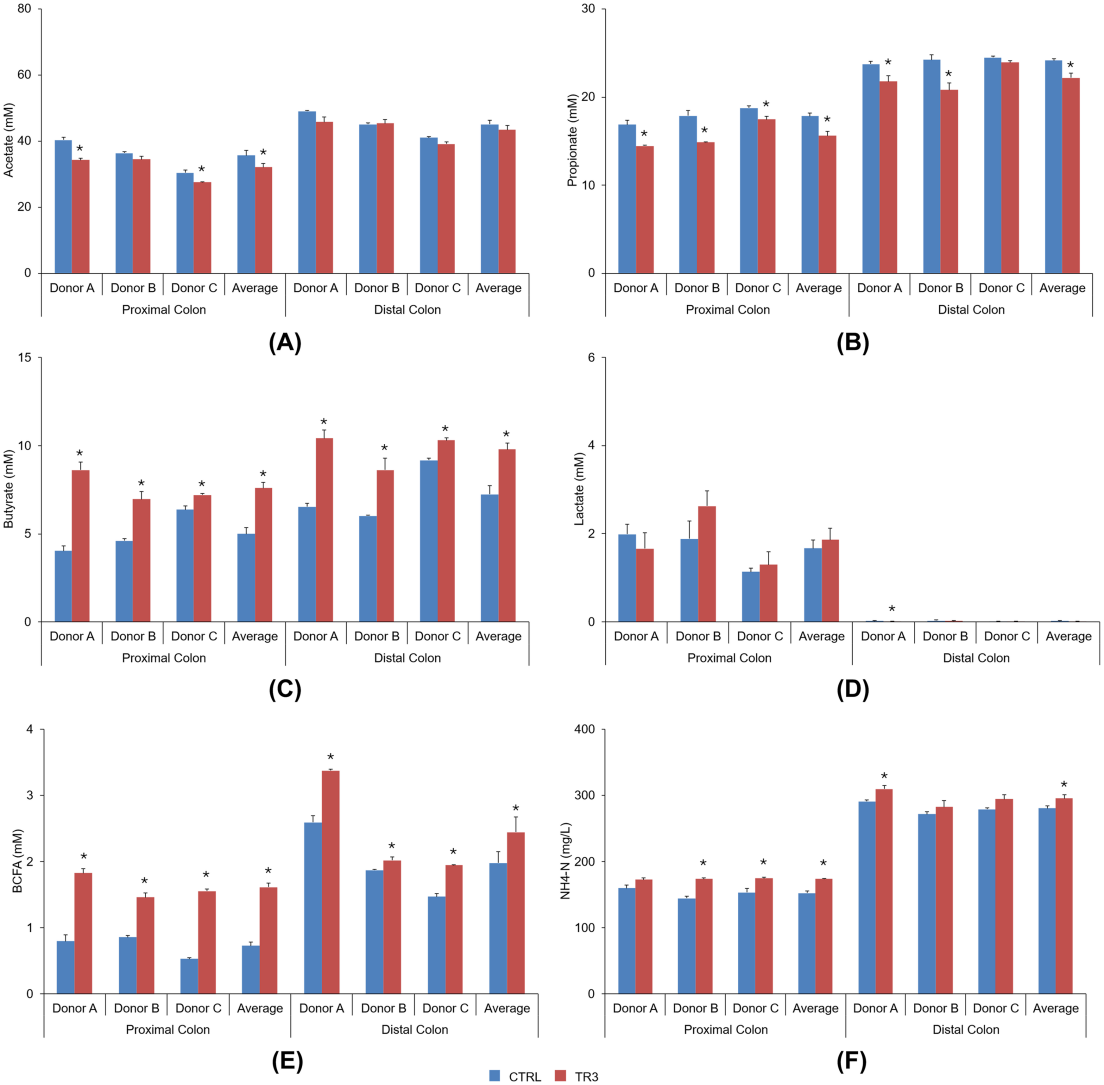
Bar graphs visualizing the level of acetate **(A)**, propionate **(B)**, butyrate **(C)**, lactate **(D)**, BCFA **(E)**, and ammonium **(F)** during the control period and after 3 weeks of daily administration of tributyrin for each individual donor and across donors in the proximal and distal colon reactors of the Triple-L-SHIME^®^ experiment. No test products were administered to the colon reactors during the control period, tributyrin was administered daily to the colon reactors during the three-week treatment period. Data are plotted as mean ± standard error of the mean (each donor, *n* = 3; average, *n* = 9). *p < 0.05 versus control. *p*-Values were determined using a two-tailed homoscedastic Student’s t-test. BCFA, branched-chain fatty acid; CTRL, control; NH4-N, ammonium; SHIME^®^, Simulator of the Human Intestinal Microbial Environment; TR3, treatment week 3.

Microbial metabolite levels during the control period and following repeated tributyrin supplementation (TR3) in the Triple-L-SHIME^®^ colonic supernatants are shown in Figure 2. For each individual donor and across donors, both the acetate and propionate levels were not stimulated by tributyrin supplementation, with similar or even significantly lower levels observed at the end of the treatment period compared to the control period in both the proximal and distal colon compartments (Figures 2A,B). In contrast, the levels of butyrate were significantly increased with tributyrin supplementation for each individual donor and across donors in both colon compartments (control vs. TR3, *p* < 0.05 for all) (Figure 2C). Lactate levels were not affected by tributyrin supplementation, with similar levels detected during the TR3 and control periods for all donors and across donors in both colon compartments (except for a significant reduction in the distal colon of donor A following product administration) (Figure 2D).

BCFA levels were significantly increased with tributyrin supplementation for all donors and across donors in both the proximal and distal colon compartments (control vs. TR3, *p* < 0.05 for all) (Figure 2E). Levels of ammonium were significantly increased with tributyrin supplementation for donors B and C and across donors in the proximal colon compartment and for donor A and across donors in the distal colon compartment (control vs. TR3, *p* < 0.05 for all) (Figure 2F).

### 3.3 Untargeted metabolomics

**Figure 3:**
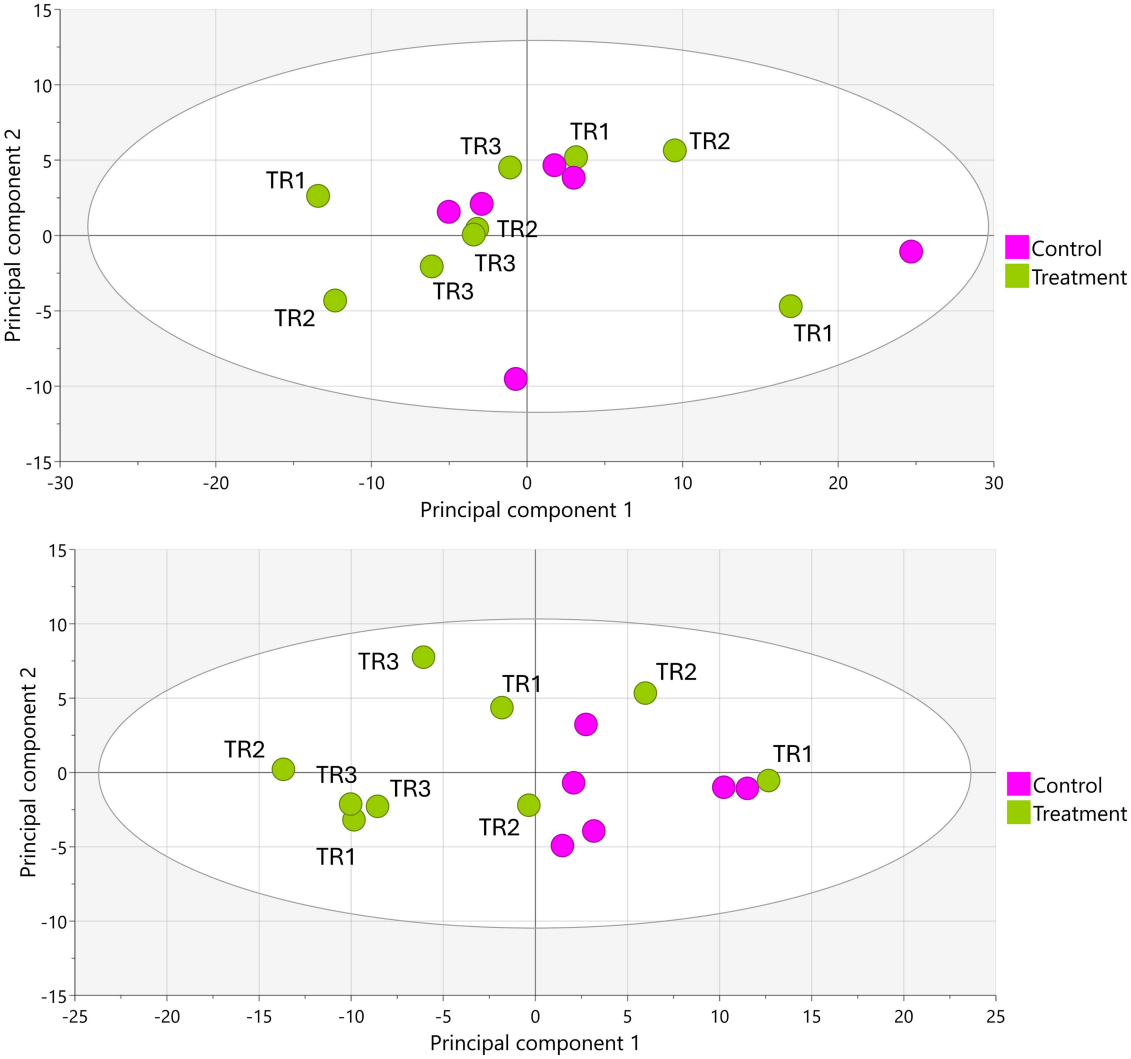
PCA-X score plots based on LA-REIMS data (negative ionization mode) obtained using biological samples (*n* = 15) from the proximal colon (top) and distal colon (bottom) during the Triple-L-SHIME^®^ experiment. No test products were administered to the colon reactors during the control period, tributyrin was administered daily to the colon reactors during the three-week treatment period. Samples were collected from the indicated colon reactor during the control period (*n* = 6), TR1 (*n* = 3), TR2 (*n* = 3), and TR3 (*n* = 3) and subjected to LA-REIMS. LA-REIMS, Laser-Assisted Rapid Evaporative Ionization Mass Spectrometry; PCA-X, unsupervised principal component analysis; SHIME^®^, Simulator of the Human Intestinal Microbial Environment; TR1, treatment week 1; TR2, treatment week 2; TR3, treatment week 3.

Upon LA-REIMS analysis, a total of 1,621 unique metabolic features were listed as constituents of the metabolic fingerprints. PCA-X and supervised OPLS-DA modeling revealed no significant metabolic impact of tributyrin supplementation in the proximal colon compartment at any timepoint (Figure 3). In the distal colon, no significant metabolic effects were observed between the control period and either the treatment week 1 (TR1) or treatment week 2 (TR2) periods; however, comparison between the control period and the TR3 period revealed significant metabolic alterations. This was demonstrated by segregation of the control and TR3 samples in PCA-X modeling (Figure 3) and a valid result for OPLS-DA modeling (control vs. TR3: Q^2^[Y], 0.709; permutation test result, passed; *p* = 0.024).

### 3.4 Metagenomic analysis

**Figure 4:**
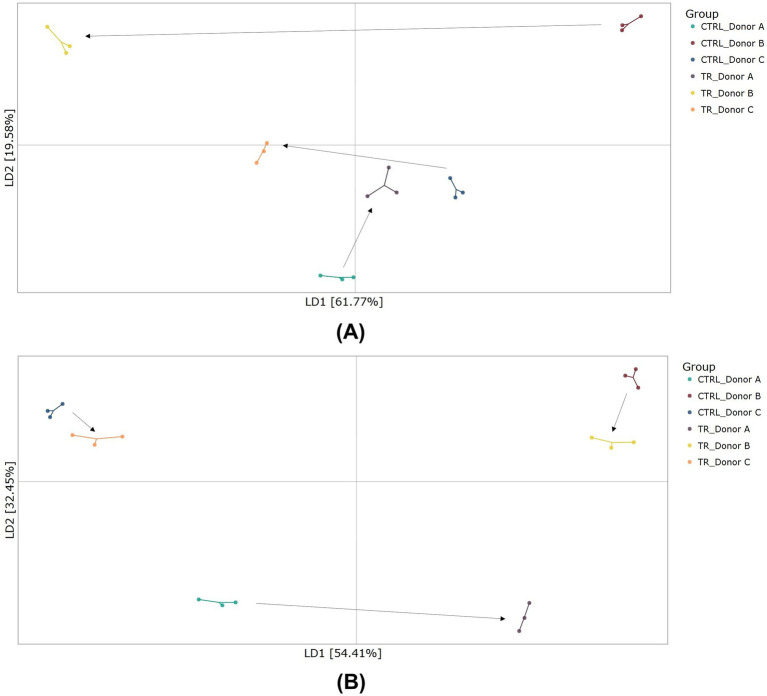
Beta-diversity as demonstrated by DAPC showing differences in community composition in the proximal colon **(A)** and distal colon **(B)** at the end of the control and treatment periods of the Triple-L-SHIME^®^ experiment. No test products were administered to the colon reactors during the control period, tributyrin was administered daily to the colon reactors during the three-week treatment period. Samples were collected at the end of the control and treatment periods from each reactor representing three human donors (*n* = 3 per donor) and subjected to metagenomics analysis. Each dot represents one sample. The arrows represent shifts between the control and treatment conditions for each donor. CTRL, control; DAPC, discriminant analysis of principal components; LD, linear discriminant; SHIME^®^, Simulator of the Human Intestinal Microbial Environment; TR, treatment.

DAPC plots to assess beta-diversity revealed a shift in the microbial communities for all donors following tributyrin supplementation in both the proximal and distal colon compartments (Figures 4A,B). The shifts were particularly pronounced in the proximal colon compartment.

**Figure 5:**
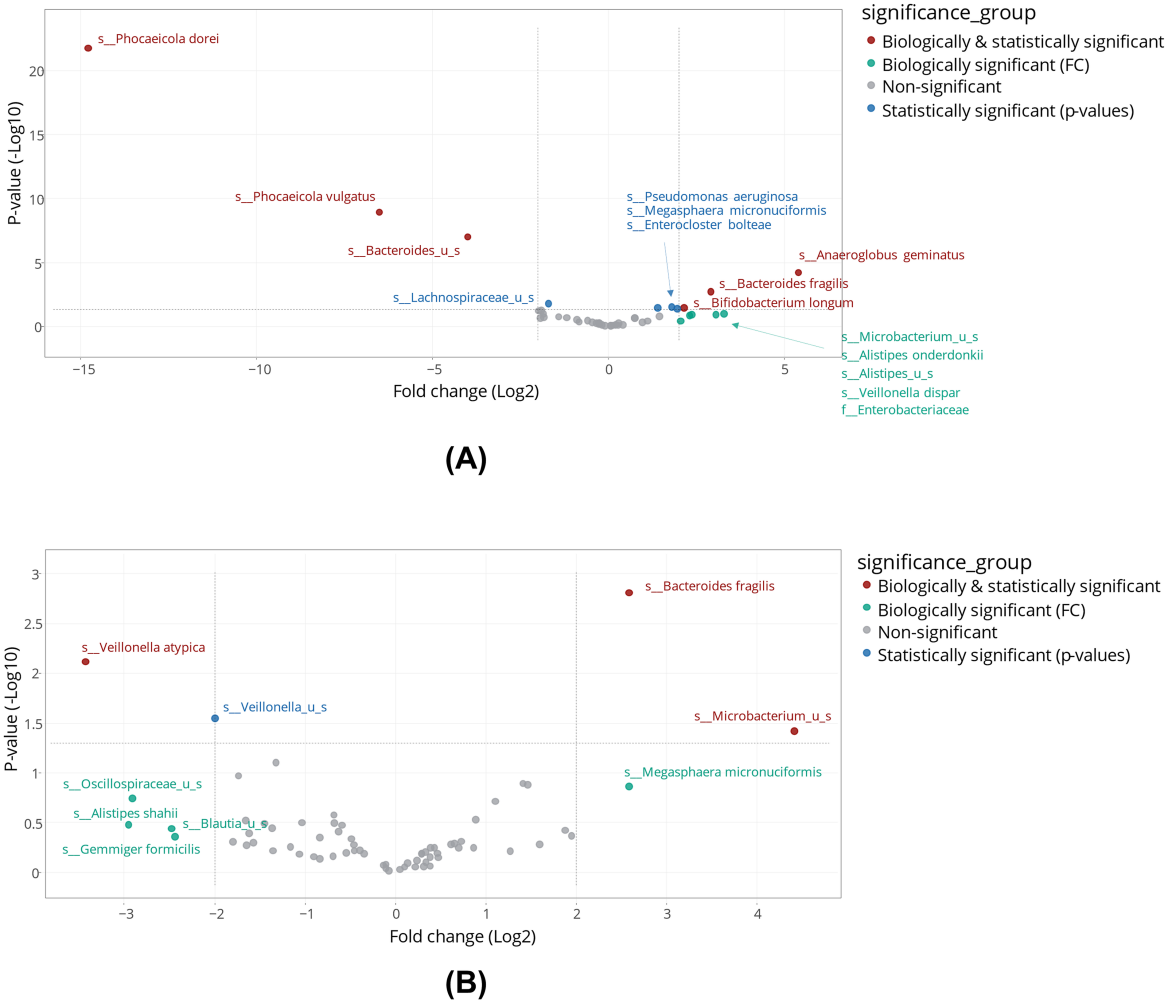
Differential abundance analysis in the proximal colon **(A)** and distal colon **(B)** obtained at the end of the control and treatment periods of the Triple-L-SHIME^®^ experiment. No test products were administered to the colon reactors during the control period, tributyrin was administered daily to the colon reactors during the three-week treatment period. Samples were collected at the end of the control and treatment periods from each reactor representing three human donors (*n* = 3 per donor) and subjected to metagenomics analysis followed by treeclimbR analysis. Statistical significance is plotted as a function of FC, classifying taxa into four categories: (1) not significant and not biologically relevant (−2 < log_2_FC < +2, and −log_10_[*p*-value] < 1.3), (2) biologically relevant, but not statistically significant (log_2_FC < −2 or log_2_FC > +2, and −log_10_[*p*-value] < 1.3), (3) statistically significant, but not biologically relevant (−2 < log_2_FC < +2, and -log_10_[*p*-value] > 1.3), and (4) biologically and statistically significant (log_2_FC < −2 or log_2_FC > +2, and -log_10_[*p*-value] > 1.3). FC, fold change; SHIME^®^, Simulator of the Human Intestinal Microbial Environment.

As shown in the volcano plots (Figure 5), supplementation with tributyrin resulted in biologically and statistically significant enrichments of several bacterial species. In the proximal colon, levels of several primary degraders were enhanced, e.g., *Bifidobacterium longum* (biologically and statistically significant), *Bacteroides fragilis* (biologically and statistically significant, with similar effects in the distal colon), and several *Alistipes* spp. (biologically significant) (Figure 5A). Tributyrin stimulated some butyrate-producing bacteria in an apparent positive feedback loop. Other notable bacterial enrichments included *Megasphaera micronuciformis* in both colon compartments (proximal colon, statistically significant; distal colon, biologically significant), *Anaeroglobus geminatus* and *Veillonella dispar* in the proximal colon compartment (biologically and statistically significant, and biologically significant, respectively), and unspecified *Microbacterium* spp. in both colon compartments (proximal colon, biologically significant; distal colon, biologically and statistically significant) (Figures 5A,B). Statistically reduced microbial species following product supplementation included *Phocaeicola dorei*, *Phocaeicola vulgatus* and unspecified *Bacteroides* spp. in the proximal colon, and *Veillonella atypica* and unspecificied *Veillonella* spp. (the latter not reaching biological significance) in the distal colon.

**Figure 6:**
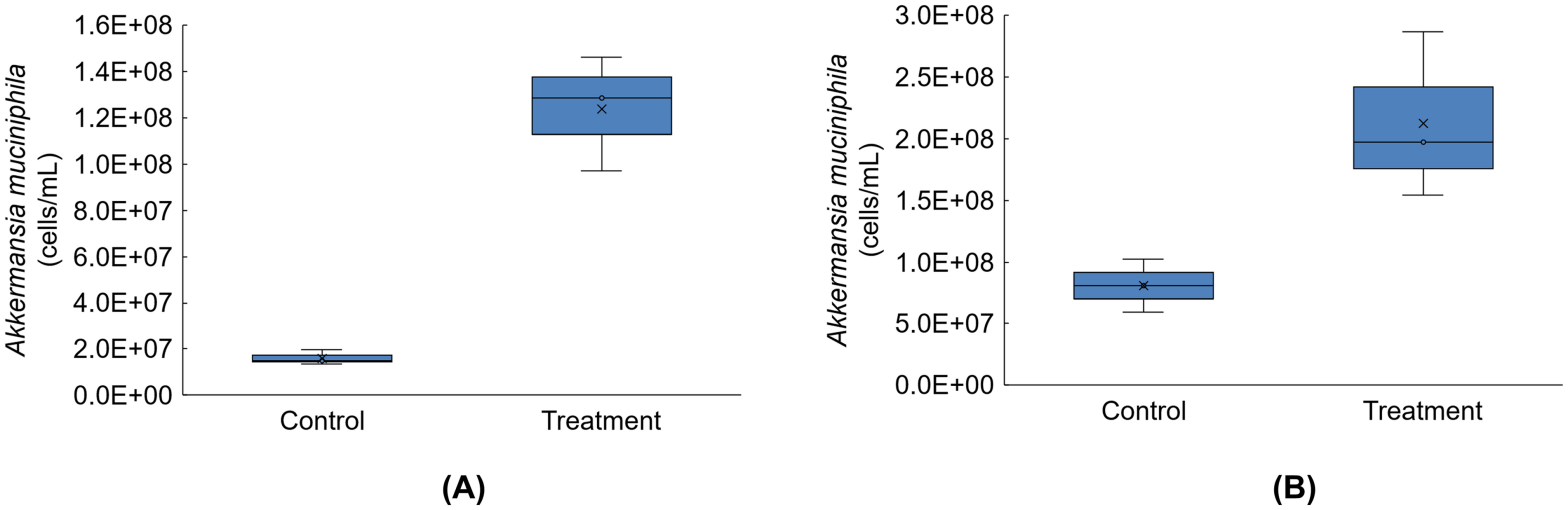
Boxplots showing absolute abundances (cells/mL) for *Akkermansia muciniphila* for donor A **(A)** and donor C **(B)** in the distal colon at the end of the control and treatment periods of the Triple-L-SHIME^®^ experiment (levels of this taxon were below the limit of quantification for donor B). No test products were administered to the colon reactors during the control period, tributyrin was administered daily to the colon reactors during the three-week treatment period. Samples were collected at the end of the control and treatment periods from each reactor representing donor A and donor C (*n* = 3 per donor) and subjected to metagenomics analysis. ‘x’ in the boxplot indicates the average value. SHIME^®^, Simulator of the Human Intestinal Microbial Environment.

Absolute abundances of *Akkermansia muciniphila* were highly enriched in the distal colon compartments for donors A and C following tributyrin supplementation (Figure 6); *Akkermansia muciniphila* levels were below the limit of detection for donor B (data not shown).

### 3.5 Intestinal permeability and cytokine responses

**Figure 7:**
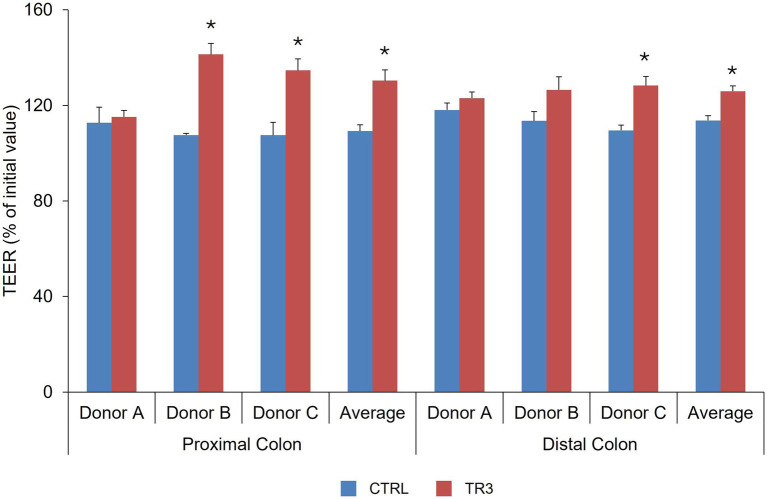
Effect of colonic suspensions on TEER of the Caco-2/THP1 co-cultures. TEER was measured 24 h after treatment of the co-cultures with colonic supernatants collected from the Triple-L-SHIME^®^ experiment at the end of the control and treatment periods. Data are plotted as mean ± standard deviation. *p < 0.05 for the treatment versus the control period. *p*-Values were determined using a two-way ANOVA with Sidak’s multiple comparisons test. ANOVA, analysis of variance; CTRL, control; SHIME^®^, Simulator of the Human Intestinal Microbial Environment; TEER, transepithelial electrical resistance; TR3, treatment week 3.

The increase in TEER value following LPS exposure (% of initial value) was significantly higher versus control with tributyrin-supplemented proximal colonic supernatants for donors B and C and across donors, and with tributyrin-supplemented distal colon supernatants for donor C and across donors (control vs. TR3, *p* < 0.05) (Figure 7).

**Figure 8:**
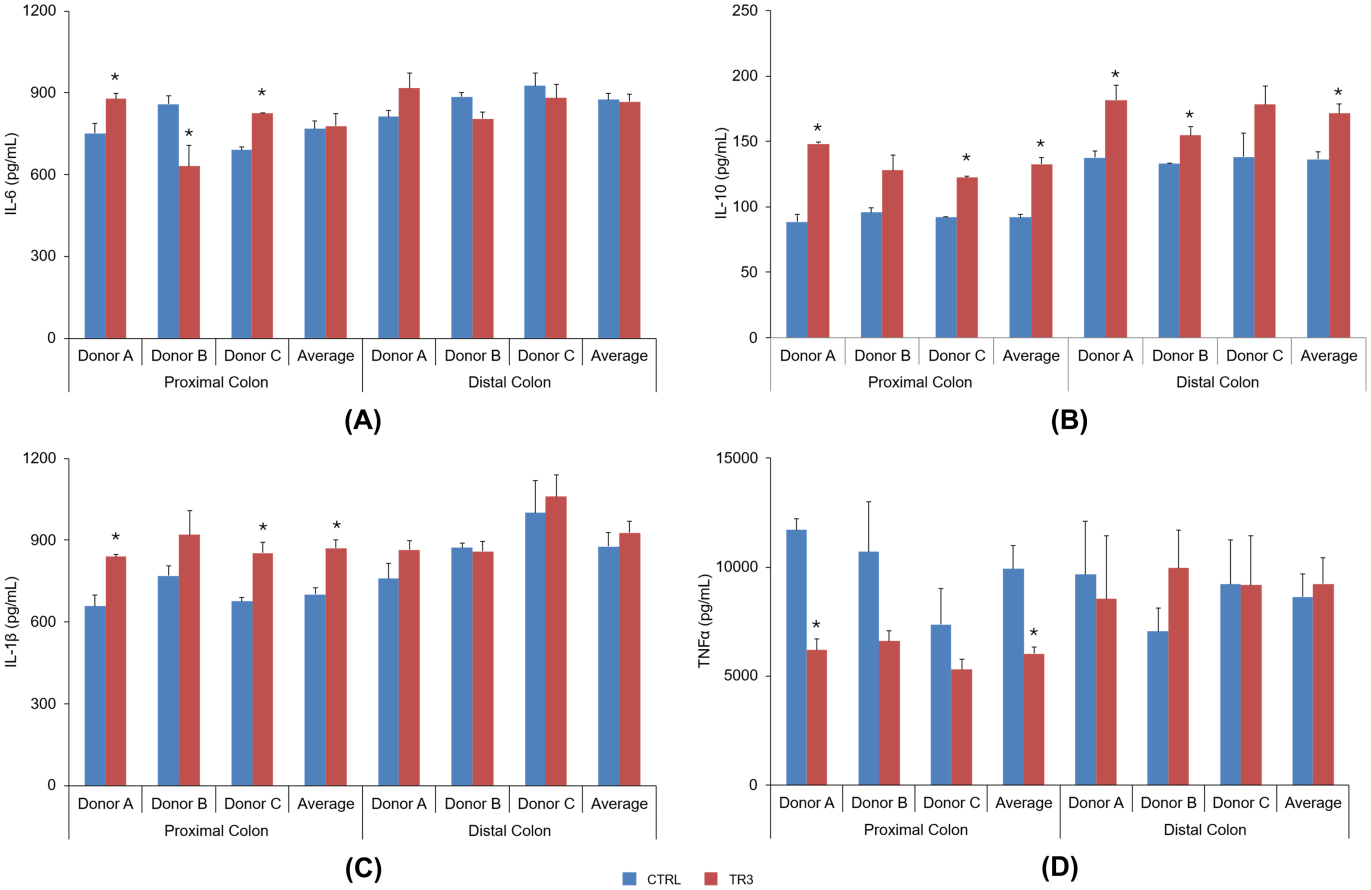
Effect of colonic suspensions on IL-6 **(A)**, IL-10 **(B)**, IL-1β **(C)**, and TNF-α **(D)**. Cytokine levels were measured 6 h after LPS treatment on the basolateral side of the Caco-2/THP1 co-cultures after pre-treatment of the apical side for 24 h with the colonic suspensions collected from the Triple-L-SHIME^®^ experiment. Data are plotted as mean ± standard error of the mean. *p < 0.05 for the treatment versus the control period. *p*-Values were determined using a two-way ANOVA with Sidak’s multiple comparisons test. ANOVA, analysis of variance; CTRL, control; IL, interleukin; LPS, lipopolysaccharide; SHIME^®^, Simulator of the Human Intestinal Microbial Ecosystem; TNF, tumor necrosis factor; TR3, treatment week 3.

In the Caco-2/THP co-culture model, IL-6 levels following exposure to colonic supernatants from the control and TR3 periods were not consistent among donors for both proximal and distal colon supernatants, thus not reaching any statistical significance across donors (Figure 8A). IL-10 levels were significantly increased with tributyrin-supplemented supernatants from both the proximal colon (donors A and C and across donors; control vs. TR3, *p* < 0.05) and distal colon (donors A and B and across donors; control vs. TR3, *p* < 0.05) (Figure 8B). IL-1β levels were significantly increased with tributyrin-supplemented proximal colon supernatants (donors A and C and across donors; control vs. TR3, *p* < 0.05) but not with distal colon supernatants (Figure 8C). Levels of TNF-α were significantly decreased with tributyrin-supplemented proximal colon supernatants from donor A and across donors (control vs. TR3, *p* < 0.05) and no significant differences were observed with tributyrin-supplemented distal colon supernatants (Figure 8D).

## 4 Discussion

This study evaluated the effects of tributyrin (CoreBiome®) supplementation on the metabolic activity and community composition of the gut microbiome from three healthy donors using the Triple-L-SHIME^®^ model. Effects on intestinal barrier integrity and host immune response were also assessed. We found that tributyrin supplementation increased levels of butyrate and caused a shift in the microbial community, with enrichments in several beneficial bacterial species. Metabolic impacts were observed later in the treatment period, demonstrating the importance of long-term administration. Evaluation of host microbiome interactions revealed that tributyrin-supplemented colonic fermentations provided protection from inflammation-induced intestinal barrier disruption and modulated cytokine secretion, increasing IL-10 levels and decreasing TNF-α levels.

The upper GIT simulation demonstrated that while some tributyrin was hydrolyzed to butyrate in the small intestine, over half of the initial dose was available to transit to the colon. Indeed, tributyrin supplementation strongly increased butyrate levels in both the proximal and distal colon compartments in the Triple-L-SHIME^®^ experiment. Considering the 1:3 conversion ratio of tributyrin to butyrate (i.e., one molecule of tributyrin is hydrolyzed into three molecules of butyrate), the observed increase in butyrate levels was most likely a direct result of the metabolism of tributyrin to butyrate by the gut microbiota.

Untargeted metabolomic analysis revealed significant metabolic alterations in the distal colon after 3 weeks of tributyrin supplementation, which were not observed at TR1 or TR2. This suggests that prolonged administration of tributyrin is necessary to achieve maximal effects on microbial metabolism. Future studies utilizing ultra-high-performance liquid chromatography-high resolution mass spectrometry to identify the specific metabolites that are enriched with tributyrin supplementation will build upon this finding.

Beta-diversity analysis showed that tributyrin supplementation resulted in a shift in the microbial community composition for all donors in both colonic compartments, with stronger effects observed in the proximal colon. Specifically, several primary substrate degraders were enriched with tributyrin supplementation (*Bifdobacterium longum*, *Bacteroides fragilis*, *Alistipes* spp.), mainly in the proximal colon, confirming previous findings (18, 23). Primary degraders break down complex carbohydrates, producing SCFAs and other bacterial metabolites that have beneficial effects on the host (42). Additionally, *Bifidobacterium longum* has been studied as a probiotic (43) and has demonstrated health-promoting effects such as lowering cholesterol and reducing inflammation in a high fat diet mouse model (44), and improving cognitive function in older adults (45). An increase in the abundance of *Veillonellaceae* family members was also observed with product supplementation, which could be attributed to a significant enrichment of *Megasphaera micronuciformis* and *Anaeroglobus geminatus*. *Megasphaera micronuciformis*, a lactate consumer and propionate producer, has been negatively associated with total cholesterol and fasting glucose levels in healthy individuals (46), while *Anaeroglobus geminatus* is a potent butyrate producer, also able to produce acetate and propionate following substrate fermentation (47). Finally, tributyrin induced an enrichment of *Akkermansia muciniphila* in the distal colon for two of the three healthy donors tested. *Akkermansia muciniphila* produces acetate and propionate, and its abundance is inversely correlated with several diseases including inflammatory bowel disease, diabetes, and obesity (48). Preclinical models have suggested several beneficial effects of *Akkermansia muciniphila* on host metabolism (49). For example, a study in mice demonstrated that *Akkermansia muciniphila* improved the metabolic profile of obese mice fed a high-fat diet and reduced high-fat-diet-induced metabolic disorders (50). It was recently reported that *Akkermansia muciniphila* produces a glucagon-like-peptide-1 inducing protein that improves glucose homeostasis and has beneficial effects in a mouse model of metabolic disease (51). Overall, based on the above findings, it could be interesting in future studies to unravel the specific mechanism-of-action in which the lipophilic tributyrin stimulates specific saccharolytic bacterial groups in the human gut microbiome.

The intestinal barrier plays an important role in preventing harmful substances from entering the systemic circulation and potentially causing inflammation (52). Chronic disruption of the intestinal barrier is termed “leaky gut” and is associated with several health conditions, including irritable bowel syndrome, inflammatory bowel disease, autoimmune conditions, and obesity. Thus, maintaining the intestinal barrier function is important for health. Butyrate is involved in both intestinal barrier function and immunomodulation. It is a primary energy source for intestinal epithelial cells (53) and numerous studies have reported beneficial effects of butyrate on the intestinal barrier which are mediated by its effects on tight junctions, the mucus layer, and the production of antimicrobial peptides (5, 54, 55). Butyrate also has immunomodulatory effects in the colon, such as reducing levels of pro-inflammatory genes and inducing anti-inflammatory genes (55–57). Tributyrin-supplemented colonic fermentations from both the proximal and distal colon had a protective effect on inflammation-induced intestinal barrier disruption. Cytokine responses to an inflammatory signal (LPS) were also impacted by tributyrin supplementation. Secretion of the anti-inflammatory cytokine IL-10 was increased in the presence of tributyrin-supplemented proximal and distal colonic fermentations following LPS exposure. Under the same conditions, secretion of the pro-inflammatory cytokine TNF-α was decreased in the presence of proximal colon fermentations.

This study had some limitations to consider. First, while *in vitro* simulations allow for in-depth study of both the effects of test products on the gut microbiome and the mechanisms behind these effects, findings from these studies do not translate directly to *in vivo* effects. As such, further studies in humans are needed to confirm these findings, where an interesting approach could be to include ‘pure’ butyrate supplementation as a control. Second, this study included three healthy donors, which allowed for the assessment of interindividual responses to tributyrin supplementation. However, the small number of donors limits the statistical power of the analysis.

## 5 Conclusion

Over 50% of tributyrin (CoreBiome®) remained stable during upper GIT transit in both capsule and softgel formulations. Daily supplementation with tributyrin demonstrated beneficial effects to both the gut microbiome community composition and metabolic activity in the Triple-SHIME^®^ model. Further, supplementation had a protective effect on intestinal barrier integrity and demonstrated immunomodulatory effects.

## Data Availability

The raw data supporting the conclusions of this article will be made available by the authors without undue reservation.
