# Supplementary material for: Tributyrin (CoreBiome®) enhances butyrate levels and modulates the gut microbiota, barrier function, and immune response in vitro
Source: Front Nutr. 2025 Nov 24;12:1712993. doi: 10.3389/fnut.2025.1712993 (PMC12746503; doi:10.3389/fnut.2025.1712993)
Supplement: Supplementary file 1 [file Image_1.pdf]

frontiers

Supplementary Material

1 Supplementary Figures

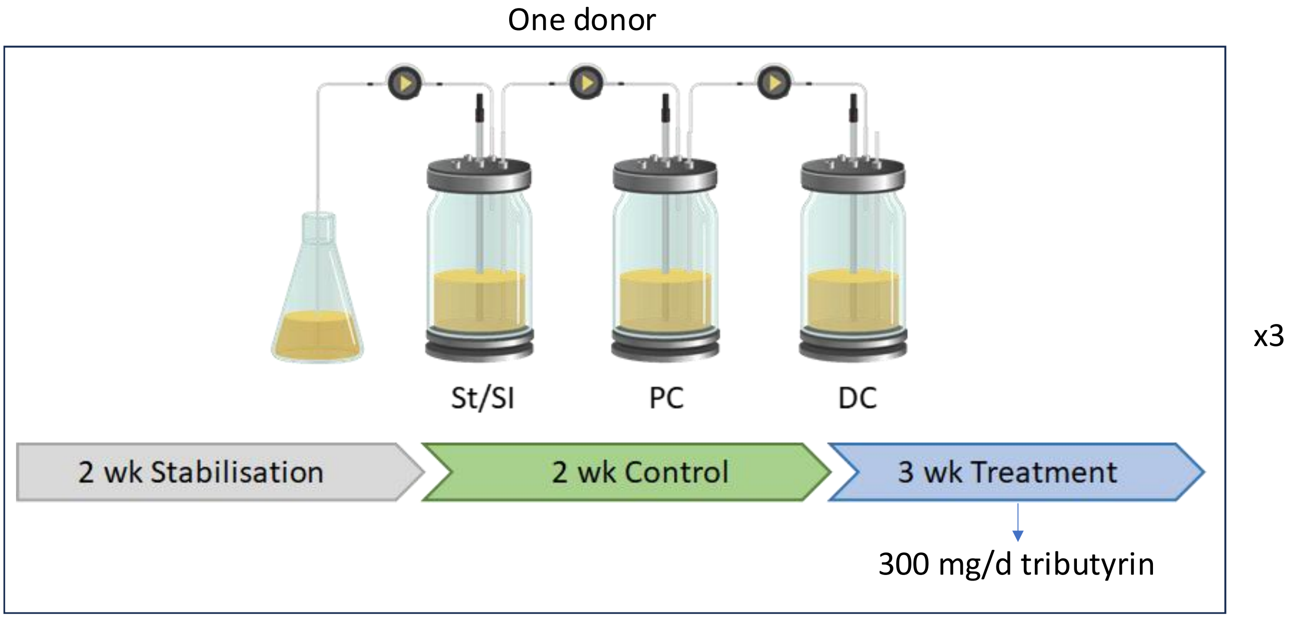

Supplementary Figure S1. Experimental design. Overview of experimental setup and timeline of the SHIME®-experiment. Tributyrin (corresponding to in vivo dose of 300 mg/d) was applied as a treatment during the treatment phase. The setup was performed for three donors, resulting in a Triple-L-SHIME® setup. St/SI: stomach/small intestine; PC proximal colon; DC: distal colon.
